# Recent advances in understanding and managing chordomas

**DOI:** 10.12688/f1000research.9499.1

**Published:** 2016-12-22

**Authors:** Carl Youssef, Salah G. Aoun, Jessica R. Moreno, Carlos A. Bagley

**Affiliations:** 1 Department of Neurosurgery, University of Texas Southwestern Medical Center, Dallas, Texas, 75390, USA

**Keywords:** chordoma, chordomas, primary bone tumours, primary bone tumors, focused particle beam radiation, targeted chemotherapy

## Abstract

Chordomas are rare primary bone tumors arising from embryonic remnants of the notochord. They are slow-growing, locally aggressive, and destructive and typically involve the axial skeleton. Genetic studies have identified several mutations implicated in the pathogenesis of these tumors. Treatment poses a challenge given their insidious progression, degree of local invasion at presentation, and high recurrence rate. They tend to respond poorly to conventional chemotherapy and radiation. This makes radical resection the mainstay of their treatment. Recent advances in targeted chemotherapy and focused particle beam radiation, however, have improved the management and prognosis of these tumors.

## Introduction

Chordomas are primary bone tumors typically involving the axial skeleton. They are rare and account for only 1% to 4% of all primary malignant bone tumors; they have an incidence of 300 newly diagnosed cases every year ^1, 2^. Presentation occurs at an older age compared with other tumors of the spine (59.9 ± 17 years) ^3^. Males are more affected than females (59.5%), and Caucasians and Hispanics are four times more prone to the disease than African-Americans ^1^. Although these tumors were previously thought to arise more frequently in the sacrum, a recent US population-based database study showed that tumor distribution was relatively even between the skull base (32%), mobile spine (32.8%), and the sacrum (29.2%) and that the remainder (6%) were in various extra-axial locations ^1^. Median survival rate is approximately 7 years, and 5-, 10-, and 20-year survival rates are 68%, 40%, and 13%, respectively ^1^.

## Clinical features

The clinical presentation of chordomas varies with tumor location, since these lesions are slow-growing and can be locally destructive. Cranial tumors frequently present initially with headaches and diplopia. Symptoms can evolve to include cranial nerve palsies, visual loss, seizures, extremity weakness or numbness, neck pain, nasal congestion, or dysphagia as adjacent structures are progressively infiltrated or compressed ^4^. On the other hand, chordomas of the spine and sacrococcygeal region usually present with local pain or with signs of neural compression, such as radiculitis, bowel or urinary incontinence, or sexual dysfunction. In advanced cases of sacrococcygeal disease, the mass can be palpated during rectal or gynecologic examination ^5, 6^.

In addition to classic clinical features, imaging plays an important role in establishing a correct diagnosis. Chordomas often appear on plain films as a destructive bone lesion with associated soft tissue calcification ^7, 8^. In contrasted computed tomography (CT) scans, chordomas usually show as a contrast-enhancing, lytic, or mixed bony lesion. They often have a myxoid component that is seen as a well-demarcated soft tissue mass containing areas of hypodensity. Their overall density is comparable to that of muscle, and calcifications are usually present, particularly in the sacrococcygeal area ^9, 10^. On magnetic resonance imaging (MRI), chordomas are hypointense or isointense on T1-weighted sequences and hyperintense on T2-weighted sequences. They tend to enhance heterogeneously with gadolinium, creating a lobulated “honeycomb” appearance ^11^. This is due to fluid, including gelatinous mucoid substance, old hemorrhage, or areas of necrosis within the tumor. Unlike osteosarcomas and chondrosarcomas of the spine, chordomas often involve the intervertebral disk as they invade adjacent vertebral segments, and they exhibit decreased or normal isotope uptake on scintigraphic studies ^5, 12^.

Despite fairly recognizable features on imaging, a timely diagnosis is often difficult to make. This is due mainly to the insidious nature of these tumors, as they frequently present with lingering chronic headaches or back pain that does not prompt patients to seek medical attention immediately ^13^. In addition, sacral lesions may go undetected on imaging, as CT and MRI scans often do not extend below the S2 level unless specifically ordered ^5^.

## Pathogenesis and genetics

Chordomas are believed to arise from embryonic remnants of the notochord. Histologically, both the notochord and chordomas consist of a nest of large, vacuolated, physaliphorous cells surrounded by an extracellular myxoid matrix ^14, 15^. The anatomic distribution of chordomas along the axial skeleton also respects the longitudinal midline axis formed by the notochord during embryonic development.

More recently, a strong genetic link corroborating the relationship between chordomas and the notochord has been established. The “T” gene, located on chromosome 6q27, encodes the “Brachyury” protein and is an evolutionarily favored gene that plays a critical role in the development of the notochord and in defining the midline of bilaterian organisms ^16^. Duplications in this gene have been found to confer major susceptibility in studies of familial chordomas, and its amplification has also been implicated in sporadic cases ^17– 20^. Interestingly, silencing of the Brachyury gene in chordoma cells *in vitro* leads to complete growth arrest and senescence ^21^. The Brachyury gene is also implicated in various human carcinomas and could be responsible for the epithelial-mesenchymal transition allowing tumors to metastasize ^22– 25^. In addition, the uniquely high levels of expression of the Brachyury protein in chordomas have allowed scientists and labs to differentiate them from other tumors of the neuroaxis, such as chondrosarcomas, with relatively high sensitivity and specificity ^26– 31^. The degree of Brachyury expression, however, has not shown a prognostic indication in chordomas ^32^.

Recent molecular analyses have revealed additional genetic abnormalities involved in the pathogenesis of chordomas. Evidence of activation of the *PI3K/AKT/TSC1/TSC2/mTOR* signaling pathway was detected in chordoma cells ^33^. On the other hand, the Stat3 pathway was found to be constitutively active in these tumors, and its level of expression was closely correlated with disease severity and survival ^34^. Not surprisingly, aberrant epidermal growth factor receptor ( *EGFR*) signaling has also been implicated in their pathogenesis ^35^. Furthermore, the activation of the *IGF1R* and loss of *MTAP*, an essential enzyme in the purine salvage pathway, were also detected ^36^. The analysis of existing chordoma cell lines has revealed additional genetic aberrations, including the loss of p16, *PTEN*, *CDKN2a/CDKN2b*, and *PDCD4* ^37, 38^. Studies of skull base lesions have implicated losses on chromosome 1p and the *FHIT* gene and gains on 1q and 2p in their pathogenesis ^39, 40^. These findings demonstrate non-random genetic alterations, in which losses are more frequent than gains, and pave the way to developing targeted treatments for these tumors ^41^.

As in other neoplasms, epigenetic alterations play an important role in understanding the tumorigenesis process, but they also provide invaluable diagnostic, prognostic, and potentially therapeutic molecular tools. Duan *et al.* provided data on microRNA (miRNA) expression in chordomas and demonstrated several miRNAs that are differentially expressed in chordoma cell lines compared with controls ^42^. The therapeutic potential of miRNAs is evident from multiple studies such as that by Zhang *et al.*, in which *EGFR*, *MET*, and *Bcl-xL* were identified as targets of two downregulated miRNAs that, when restored, were able to inhibit cell proliferation and invasion and induce apoptosis in chordoma cells ^43^. Similarly, Osaka *et al.* demonstrated that microRNA-1 is downregulated in chordoma and that its restoration suppressed not only proliferation but also migratory and invasive activities and reduced expression of the oncoprotein Slug ^44^. Further evidence on the epigenetic component of chordoma pathogenesis comes from a study by Scheipl *et al.*, in which non-selective histone deacetylase inhibitors have been shown to significantly increase apoptosis of chordoma cells *in vitro* ^45^.

## Treatment

The management of chordomas is complex and continually evolving. Despite being histologically classified as low-grade tumors, chordomas are locally invasive and highly recurrent, which makes their prognosis similar to that of more malignant lesions ^6, 46^. The slow-growing nature of these tumors and their low cellular turnover rate also render them clinically resistant to traditional chemotherapy, and they have only limited *ex vivo* response to a few agents, such as doxorubicin, Yondelis, Zalypsis, and cisplatin ^37, 47^. The use of traditional radiation therapy is also limited, as the tolerance of the spinal cord and brainstem to the doses required for clinical effectiveness is restricted ^48, 49^. For all of these reasons, the mainstay of treatment of chordomas remains aggressive surgical resection with wide surgical margins ^47, 50, 51^. However, en bloc surgical resection can be challenging because of frequent proximity to or invasion of vital neural structures. As a result, intra-lesional excision or partial debulking in order to decompress vital structures is often the only available option, which may lead to tumor seeding or significant residual tumor or both ^52^. In one study, the disease-free interval for patients undergoing radical resection for sacral chordoma was 2.27 years, compared with only 8 months for patients who underwent subtotal excision ^47^. In another study, patients who underwent surgical resection survived significantly longer than those who did not undergo resection, regardless of adjuvant therapy (151 versus 81 months) ^53^. Unfortunately, prognosis remains disappointing even with successful en bloc resection, and the disease-free interval is relatively short ^50^.

Advances in surgical techniques have allowed surgeons to approach chordomas with less invasive resections. For clival chordoma, multiple studies have demonstrated that the endoscopic endonasal approach provides an effective, yet less invasive and more direct, corridor to the clivus compared with the traditional open craniotomy and transoral techniques ^54– 57^. On the other hand, surgical resection (posterior only or combined anterior-posterior approaches) of sacral chordoma has remained invasive given the typically large size of the tumor and the degree of local invasion at the time of diagnosis. However, advances in surgical resection and reconstruction techniques have allowed for more preservation of nerve roots and decreases in operative time and blood loss ^58– 60^. In the case of chordomas of the mobile spine, newer techniques, such as complete spondylectomy, have been developed to widen the margin of resection and to decrease local recurrence ^61, 62^.

The main role of radiation therapy in the management of chordomas has been as an adjuvant treatment to surgery. Residual disease after surgery rarely regresses and often leads to progression, regardless of the radiation dose delivered ^63^. However, recent advances in particle beam therapy, such as proton beam and carbon ion beam, hold some promise. In a very recent study assessing proton therapy in combination with surgical resection for skull base chordomas, the reported 5- and 7-year local control rates were 75.8% and 70.9%, respectively ^64^. Another systematic review also concluded that proton therapy improves long-term local control and survival in patients with skull base chordoma ^65^. In tumors of the sacrum and spine, surgical resection coupled with proton therapy provided a 5-year overall survival and local control rate of 81% and 62%, respectively ^66^. Carbon ion beam appears to be comparable to proton therapy. In a study of skull base chordomas treated with surgical resection followed by carbon ion radiation, local control was reported to be as high as 80.6% and 70.0% at 3 and 5 years, respectively ^67^. In another study on the effectiveness of carbon ion therapy in patients with unresectable sacral chordomas, carbon ion therapy appeared to provide a promising alternative to surgery in slowing disease progression, offering a markedly high 89% 5-year local control rate ^68^. More recently, a study on the utility of particle beam therapy (carbon ion or protons) in patients with primary sacral chordoma also reported promising results: 3-year local control, overall survival, and progression-free survival of 94%, 83%, and 68%, respectively ^69^.

Despite the promising outcomes of particle beam radiation in the treatment of chordomas, its use is still limited; specialty centers are needed to house the machines because of their size, and treatment remains expensive ^70^. Furthermore, owing to the rarity of the disease and the difficulty in randomly assigning patients into groups other than the standard of care, there are currently no published data from randomized trials assessing these new tools ^71^. Nonetheless, a randomized trial comparing proton versus carbon ion radiation therapy in patients with sacrococcygeal chordoma is under way ^72^.

With the aforementioned challenges in the management of this complex disease, the quality of life of patients with chordoma is an integral aspect of patient management and counseling. Not surprisingly, a study of patients with chordoma in the post-treatment phase demonstrated lower quality of life compared with the general population, and the most significant determinants were neurologic deficits (for example, bowel/bladder dysfunction and sensory deficits), pain medication use, corticosteroid use, and higher levels of depression ^73^. Another study assessing quality of life during treatment with proton beam therapy demonstrated no change in quality of life before and at completion of treatment, although the long-term impact has yet to be determined ^74^.

## Future directions

The diagnosis and management of chordomas are evolving as surgical and adjuvant radiation techniques are continually being refined. The most promising treatments, however, lie in the ongoing molecular analysis of these tumors and the development of targeted therapy. Multiple trials have shown promising results with new experimental treatments. Hsu *et al.* demonstrated that silencing of the Brachyury gene can lead to the differentiation and senescence of chordoma cells ^21^. Interestingly, Heery *et al.* demonstrated partial response in advanced chordoma patients who received an immune-stimulating therapeutic cancer vaccine designed to elicit Brachyury-specific T-cell responses ^75^. Yang *et al.* reported growth inhibition in different chordoma cell lines after application of *SD-1029*, an inhibitor of Stat3 activation ^34^. The detection of *MTAP* deficiency in some chordomas by Sommer *et al.* ^36^ has introduced the possibility of targeting those *MTAP*-deficient chordomas with selective *de novo* purine synthesis inhibitors, a strategy that has been applied successfully to other types of *MTAP*-deficient tumors ^76^. Presneau *et al.* identified the activation of the *AKT/mTOR* pathway in the majority of chordomas, suggesting a potential therapeutic role for combined *AKT* and *mTOR* inhibitors, such as rapamycin or its analogues ^33^. Another target, PDGFR, has also been implicated in chordoma, and multiple studies on imatinib mesylate (alone or when combined with sirolimus) have shown antitumor activity in patients with chordoma ^77– 80^. A phase II trial by the French Sarcoma Group studying sorafenib in locally advanced and metastatic chordoma has also shown promising results ^81^. Similarly, Shalaby *et al.* reported *EGFR* as a potential therapeutic target as aberrances in its signaling have been implicated in chordoma pathogenesis, arguing for a potential role of *EGFR* inhibitors in the management of some chordomas ^35^.

These findings represent a collective effort to expand our understanding of chordomas and to identify molecular targets that will allow the creation of more efficient and potentially personalized treatments. They also highlight the importance of an interdisciplinary collaboration between surgeons and scientists in an effort to continually improve our management of this complex disease.
